# Vibrational and DFT Studies and Anticancer Activity of Novel Pd(II) and Pt(II) Complexes with Chloro Derivatives of 7-Azaindole-3-Carbaldehyde

**DOI:** 10.3390/molecules29245909

**Published:** 2024-12-14

**Authors:** Ksenia Szmigiel-Bakalarz, Dagmara Kłopotowska, Joanna Wietrzyk, Magdalena Malik, Barbara Morzyk-Ociepa

**Affiliations:** 1 Institute of Chemistry, Faculty of Science and Technology, Jan Dlugosz University in Czestochowa, Armii Krajowej 13/15, 42-200 Czestochowa, Poland; kseniaszmigiel@interia.pl; 2 Hirszfeld Institute of Immunology and Experimental Therapy, Polish Academy of Sciences, 12 Rudolf Weigl St., 53-114 Wroclaw, Poland; dagmara.klopotowska@hirszfeld.pl (D.K.); joanna.wietrzyk@hirszfeld.pl (J.W.); 3 Faculty of Chemistry, Wroclaw University of Science and Technology, Wybrzeże Wyspiańskiego 27, 50-370 Wroclaw, Poland; magdalena.malik@pwr.edu.pl

**Keywords:** 4-chloro-7-azaindole-3-carbaldehyde, 5-chloro-7-azaindole-3-carbaldehyde, platinum(II) complexes, palladium(II) complexes, IR spectroscopy, Raman spectroscopy, DFT, antiproliferative activity

## Abstract

This study investigates the structural, vibrational, and biological properties of novel palladium(II) and platinum(II) complexes with 5-chloro-7-azaindole-3-carbaldehyde (5ClL) and 4-chloro-7-azaindole-3-carbaldehyde (4ClL) ligands. Infrared and Raman spectroscopy, combined with DFT (ωB97X-D) calculations, provided valuable information about metal–ligand interactions, the *cis* or *trans* conformation of the aldehyde group in the ligands, and the presence of *trans* isomers in the metal complexes obtained in the solid state. *In vitro* tests were used to evaluate the antiproliferative activity of the novel complexes against several cancer cell lines, including ovarian cancer (A2780), cisplatin-resistant ovarian cancer (A2780cis), colon cancer (HT-29), and triple-negative breast cancer (MDA-MB-231), as well as normal mouse fibroblasts (BALB/3T3). The platinum complex, *trans*-[PtCl_2_(5ClL)_2_], exhibited superior activity against A2780cis (IC_50_ = 4.96 ± 0.49 µM) and MDA-MB-231 (IC_50_ = 4.83 ± 0.38 µM) compared to cisplatin, while the palladium complexes (*trans*-[PdCl_2_(4ClL)_2_] and *trans*-[PdCl_2_(5ClL)_2_]) demonstrated enhanced selectivity with reduced toxicity to normal fibroblasts (IC_50_ = 11.29 ± 6.65 µM and 14.98 ± 5.59 µM, respectively).

## 1. Introduction

The development of metal-based anticancer agents has long been a central focus in medicinal chemistry, with platinum(II) and palladium(II) complexes playing a pivotal role in this research [1,2,3,4,5]. Cisplatin, the first FDA-approved platinum-based drug, revolutionized cancer treatment, especially for testicular and ovarian cancers. However, its clinical use is limited by severe side effects, drug resistance, and ineffectiveness against certain types of cancer. These limitations have driven ongoing research into new platinum(II) and palladium(II) complexes with improved therapeutic profiles and reduced toxicity [6,7,8,9,10,11,12].

One promising strategy to enhance the biological activity of these metal complexes is the incorporation of heterocyclic ligands, particularly those with nitrogen-rich structures [13,14,15]. Among them are the azaindole derivatives, which have gained attention for their ability to modify the properties of metal complexes, improving both cytotoxicity and selectivity [16,17]. Platinum(II) complexes with halogeno-substituted 7-azaindoles (L), such as *cis*-[PtCl_2_(L)_2_], have revealed greater cytotoxicity than cisplatin in some cancer cell lines [18,19,20]. In contrast, the corresponding palladium(II) complexes, specifically *trans*-[PdCl_2_(L)_2_], showed a lower cytotoxicity compared to cisplatin, as reflected by their IC_50_ values [21].

In our previous studies, we investigated the biological activity of platinum(II) and palladium(II) complexes with 7-azaindole-3-carbaldehyde (7AI3CA) [22,23]. The new complex, *trans*-[PtCl_2_(7AI3CA)_2_], exhibited cytotoxicity similar to that of cisplatin in LoVo (colon cancer) and MCF7 (breast cancer) cell lines, while its cytotoxicity was significantly lower in A549 (lung cancer) compared to cisplatin. Additionally, the platinum complex was found to be approximately eight times less toxic than cisplatin against normal fibroblast cells (BALB/3T3), suggesting a potential advantage in anticancer treatment in terms of reduced side effects [22]. Moreover, the *trans*-[PdCl_2_(7AI3CA)_2_] complex demonstrated notable cytotoxicity against T47D breast cancer cell lines, with an IC_50_ value of 4.77 ± 1.61 μM, which means that it was over three times more potent than cisplatin (IC_50_ = 16.3 ± 3.9 μM) [23].

**Figure 1:**
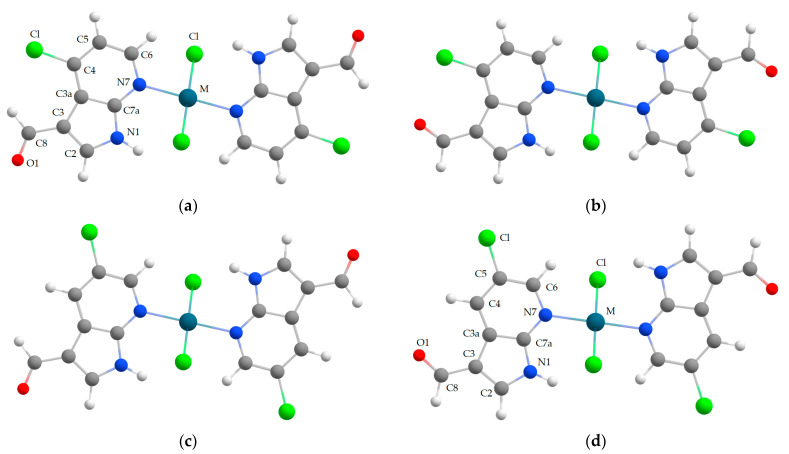

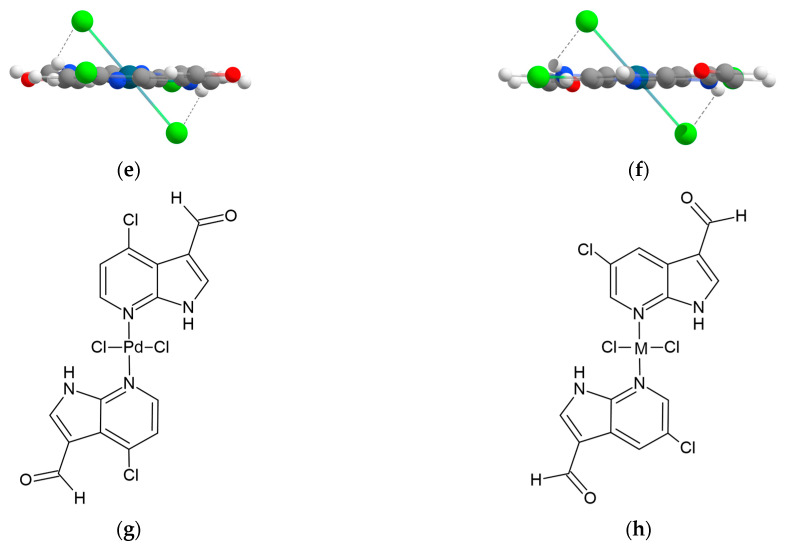
The four computational models used in the calculations are as follows: (**a**) *trans*-[PdCl_2_(4ClL)_2_] constructed from two *cis* conformers of 4ClL; (**b**) *trans*-[PdCl_2_(4ClL)_2_] constructed from two *trans* conformers of 4ClL; (**c**) *trans*-[MCl_2_(5ClL)_2_] constructed from two *cis* conformers of 5ClL; and (**d**) *trans*-[MCl_2_(5ClL)_2_] constructed from two *trans* conformers of 5ClL. Intramolecular N1–H···Cl hydrogen bonds (gray dashed lines) in the most stable models of *trans*-[PdCl_2_(4ClL)_2_] (**a**) and *trans*-[MCl_2_(5ClL)_2_] (**d**) are depicted in (**e**) and (**f**), respectively. Structural formulas for the most stable models are shown in (**g**,**h**). M = Pd(II) or Pt(II). The terms *cis* and *trans* conformers refer to the relative orientation of the C8=O1 (carbonyl) and C3=C2 (pyrrole) bonds along the exocyclic C3–C8 bond in the conformers. The atom numbering scheme is indicated for the more stable structures. Color legend: green-blue—palladium or platinum; red—oxygen; blue—nitrogen; green—chlorine; dark gray—carbon; light gray—hydrogen.

In this study, we synthesized and characterized palladium(II) and platinum(II) complexes with 4-chloro-7-azaindole-3-carbaldehyde (4ClL) and 5-chloro-7-azaindole-3-carbaldehyde (5ClL), as shown in Figure 1a and d, respectively. Our main goal was to assess whether introducing chlorine atoms at the 4 or 5 positions of 7-azaindole-3-carbaldehyde could enhance the antiproliferative properties of the palladium(II) and platinum(II) complexes. The compounds were thoroughly investigated using vibrational spectroscopy combined with theoretical calculations. Over the past decades, the density functional theory (DFT) has emerged as a powerful tool in the investigation of molecular structures and vibrational spectra of various compounds [24,25,26,27,28,29,30,31,32,33,34,35]. The DFT computations have been of great assistance to spectroscopic measurements. In particular, a good agreement between the calculated and experimental vibrational frequencies and IR/Raman intensities is essential for making unequivocal assignment of experimental spectra. Moreover, good agreement between the simulated and experimental spectra indicates that the calculated molecular geometry is correct. Thus, it is often necessary to combine experimental and theoretical methods for elucidating the molecular structures of compounds.

We also evaluated the anticancer activity of the new palladium(II) and platinum(II) complexes against a panel of human cancer cell lines. The results provide valuable insights into the impact of chloro-derivatives of 7-azaindole-3-carbaldehyde on the cytotoxic properties of metal complexes, which can be important for the future design of new metal-based anticancer drugs.

## 2. Results and Discussion

### 2.1. Molecular Structures of Trans-[PdCl_2_(4ClL)_2_] and Trans-[MCl_2_(5ClL)_2_], Where M = Pd(II) or Pt(II)

The theoretical structural analysis of the investigated metal complexes of 4-chloro and 5-chloro derivatives of 7-azaindole-3-carbaldehyde (*trans*-[PdCl_2_(4ClL)_2_] and *trans*-[MCl_2_(5ClL)_2_], where M = Pd(II) or Pt(II)) provides important information about their relative stability. In these complexes, the metal ion is coordinated by two chloro-substituted ligands (4ClL or 5ClL) through the N7 nitrogen atoms, adopting an anti-conformation relative to each other, as depicted in Figure 1a–d. The two ligands (4ClL or 5ClL) in each complex are nearly coplanar. The M–Cl bonds are positioned above and below the molecular plane in *trans* configuration. These bonds are slightly tilted with respect to the plane, as shown in Figure 1e,f. The calculated Cl–M–N7–C6 torsional angle ranges from 46.6° (for *trans*-[PdCl_2_(5ClL)_2_], Figure 1a) to 49.1° (for *trans*-[PtCl_2_(5ClL)_2_], Figure 1c). Such a tilt is caused by the formation of two intramolecular N1–H⋯Cl hydrogen bonds. The donor–acceptor distance (N1⋯Cl) varies between 2.306 Å (for *trans*-[PdCl_2_(4ClL)_2_], Figure 1b) and 2.407 Å (for *trans*-[PtCl_2_(5ClL)_2_], Figure 1c). It should be noted that intramolecular N1–H⋯Cl interactions are absent in the crystal structure of *trans*-[PtCl_2_(7AI3CA)_2_] because in the crystal, the chloride ligand is involved in an intermolecular hydrogen bond with the N–H group of the neighboring 7AI3CA ligand (the N⋯Cl distance is 3.245(13) Å) [22].

The conformational differences between the complexes are closely related to the rotation of the aldehyde group in the ligands, as shown in Figure 1a–d. Theoretical studies have been performed for each ligand in two different conformations, which differ in the relative orientation of the carbonyl (C8=O1) and pyrrole (C3=C2) groups along the exocyclic C3–C8 bond. This orientation plays a key role in determining the stability of the resulting structures of metal complexes. Similar DFT calculations were reported earlier for the free ligands 4ClL and 5ClL [24].

For *trans*-[PdCl_2_(4ClL)_2_], two structural isomers were compared: one constructed from the *cis* conformers of 4ClL (Figure 1a) and the other containing the *trans* ligand conformers (Figure 1b). Energy calculations indicate that the isomer based on the *cis* conformers is energetically more stable, with an energy difference (Δ) of approximately 4.66 kcal/mol for the Pd(II) complex (Table S1 in the Supplementary Materials). The term (Δ) denotes the difference between two calculated energies at the minima (E_min._) of the potential energy surfaces (PES) of the two investigated structures.

After including the zero-point energy (ZPE) correction, the energy difference slightly decreases to 4.47 kcal/mol (Table S1 in the Supplementary Materials). These results suggest that the *cis* conformers of 4ClL yield a more stable structure of the Pd(II) complex (it corresponds to the geometry shown in Figure 1a). Theoretical calculations for the analogous Pt(II) complex with this ligand were not performed, as no pure *trans*-[PtCl_2_(4ClL)_2_] could be synthesized.

A similar analysis was made for the Pt(II) and Pd(II) complexes with the 5-chloro derivative (5ClL) (Figure 1c,d). In this case, the structure of metal complexes containing *trans* conformers of 5ClL (shown in Figure 1d) was found to be more stable. For *trans*-[PtCl_2_(5ClL)_2_], the energy difference between complexes formed from the *cis* and *trans* conformers of 5ClL is 4.42 kcal/mol (based on E_min._), and it decreases slightly to 4.22 kcal/mol after ZPE correction. For *trans*-[PdCl_2_(5ClL), the corresponding energy differences are nearly identical to those calculated for the Pt(II) complex (see Table S2 in the Supplementary Materials). The similar Δ values for the Pt(II) and Pd(II) complexes suggest that the metal center has a minor influence on the relative stability of the complexes.

The relatively small energy differences between the complexes containing *cis* and *trans* conformers of the ligands (ranging from about 4 to 5 kcal/mol), indicate that both forms can coexist in the gas phase under standard conditions (298.15 K, 1.0 atm). However, the presence of the specific ligand conformers in metal complexes, in the solid state, can also depend on the steric effects and intermolecular interactions.

For *trans*-[PdCl_2_(4ClL)_2_] complexes, the *cis* conformers of ligands are favored, likely due to the avoidance of destabilizing oxygen-chlorine repulsions, which may be more pronounced in the *trans* arrangement. On the contrary, for *trans*-[MCl_2_(5ClL)_2_] complexes, the *trans* conformers of the 5ClL ligands are more stable due to reduced steric effects. The most stable models of these complexes are shown in Figure 1a and d, respectively. The structural formulas of these complexes are given in Figure 1g and h, respectively (they provide a clear illustration of the location of single and double bonds within the ligands). The calculated bond lengths and angles for the most stable forms of the investigated complexes are presented in Table S3 in the Supplementary Materials. Both the calculated N7-M-N7 and Cl-M-Cl bond angles are exactly 180.00°, indicating that the metal is located at a center of symmetry. These theoretical results are in very good agreement with the crystallographic data for *trans*-[PtCl_2_(7AI3CA)_2_] [22].

It is worth mentioning that preliminary studies included similar calculations for the analogous *cis* isomers of the Pd(II) and Pt(II) complexes (*cis*-[PdCl_2_(4ClL)_2_] and *cis*-[MCl_2_(5ClL)_2_], where M = Pd(II) or Pt(II)), but these complexes turned out to be less stable than the *trans* isomers. Furthermore, a single crystal X-ray diffraction and vibrational spectroscopic studies of a similar platinum(II) complex with 7-azaindole-3-carbaldehyde (7AI3CA) confirmed that it is the *trans*-[PtCl_2_(7AI3CA)_2_] isomer [22].

### 2.2. Influence of Aldehyde Group Rotation and Trans Coordination Structures on the Vibrational Spectra of Pd(II) and Pt(II) Complexes of 4ClL and 5ClL

**Figure 2:**
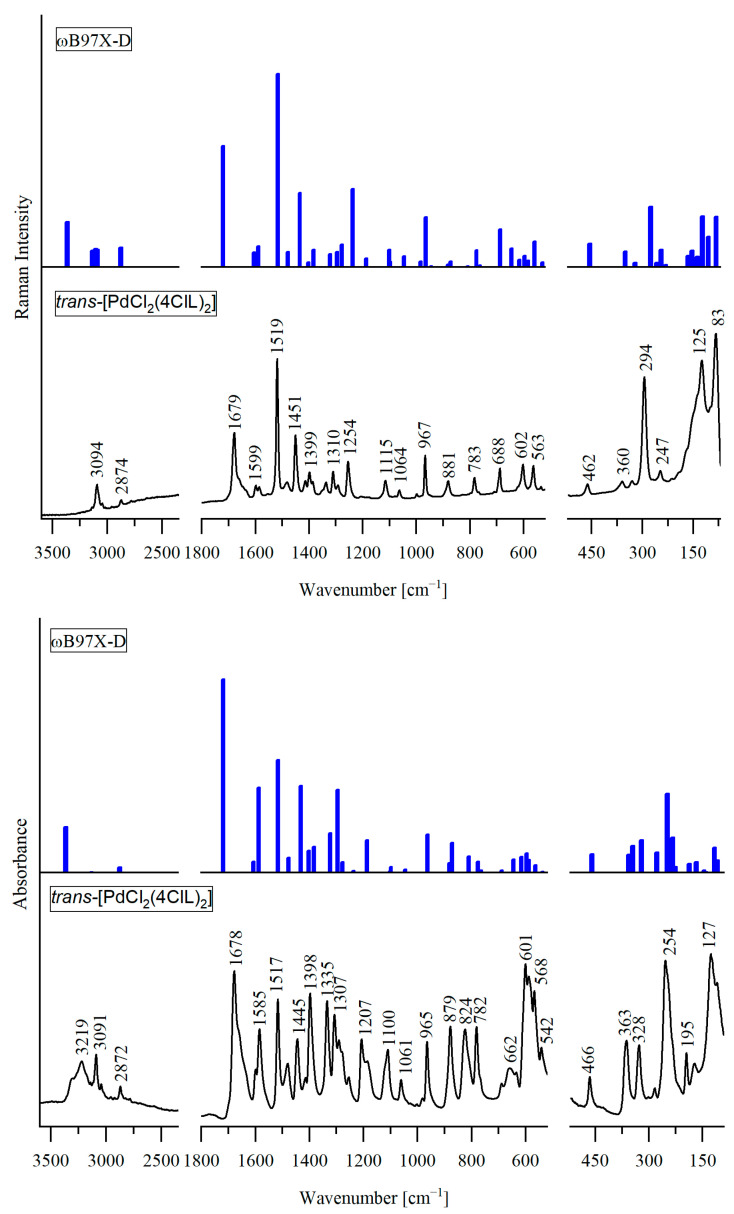
Comparison of the experimental IR (**bottom**) and Raman (**top**) spectra of *trans*-[PdCl_2_(4ClL)_2_] in the range 3600–70 cm^−1^, with the theoretical spectra calculated for the structure shown in Figure 1a using DFT method.

**Figure 3:**
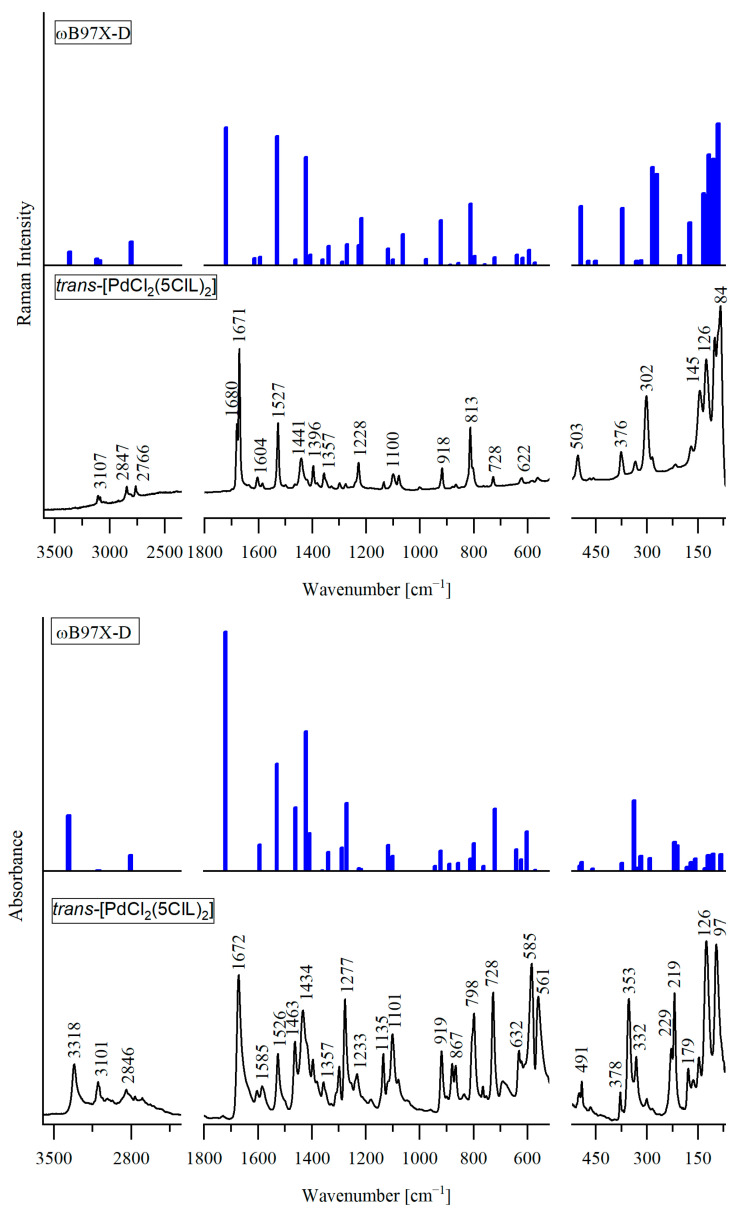
Comparison of the experimental IR (**bottom**) and Raman (**top**) spectra of trans-[PdCl_2_(5ClL)_2_] in the range 3600–70 cm^−1^, with the theoretical spectra calculated for the structure shown in Figure 1d using DFT method.

**Figure 4:**
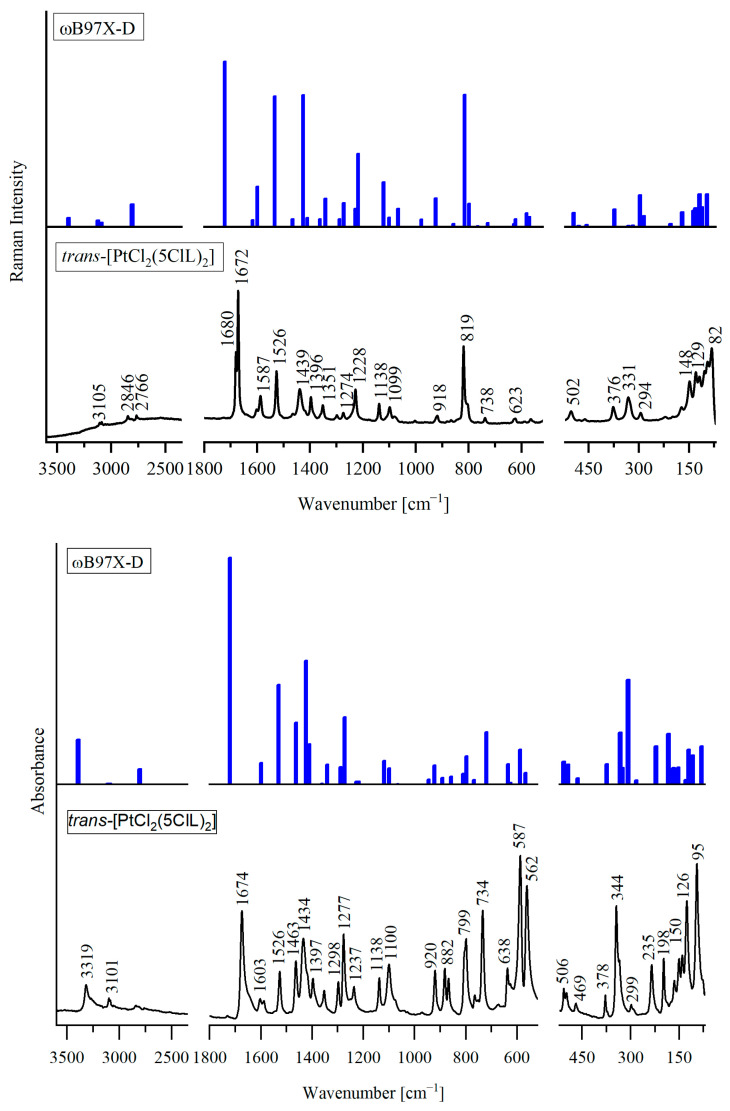
Comparison of the experimental IR (**bottom**) and Raman (**top**) spectra of *trans*-[PtCl_2_(5ClL)_2_] in the range 3600–70 cm^−1^, with the theoretical spectra calculated for the structure shown in Figure 1d using DFT method.

The experimental infrared (IR) and Raman spectra of *trans*-[PdCl_2_(4ClL)_2_], *trans*-[PdCl_2_(5ClL)_2_], and *trans*-[PtCl_2_(5ClL)_2_] are shown in Figure 2, Figure 3 and Figure 4, respectively. They match very closely to the theoretical vibrational spectra, calculated for the most stable structures presented in Figure 1a for *trans*-[PdCl_2_(4ClL)_2_] and in Figure 1d for *trans*-[MCl_2_(5ClL)_2_] (where M = Pd(II) or Pt(II)). Such a good agreement between experimental and theoretical spectra indicates that the suggested structures of the complexes are correct.

Each complex contains 37 atoms, resulting in 105 normal vibrational modes. Due to the C_i_ symmetry, there are 51 Raman active symmetric (A_g_) modes and 54 IR active antisymmetric (A_u_) modes. The detailed wavenumbers and band assignments are provided in Tables S4–S6 in the Supplementary Materials. For clarity, symmetric (g) and antisymmetric (u) vibrations with similar frequencies and assignments are combined in a single row, while distinct frequencies and assignments are presented in separate rows.

A comparison between the experimental and theoretical vibrational spectra reveals that in *trans*-[PdCl_2_(4ClL)_2_] and *trans*-[MCl_2_(5ClL)_2_] complexes, the N1H group of the pyrrole ring in the 4ClL and 5ClL ligands remains intact and does not coordinate with the Pd(II) or Pt(II) ions. In the IR spectra of these complexes, a band observed in the 3319–3219 cm^−1^ range is attributed to the antisymmetric stretching vibration, ν_as_(N1H), of the pyrrole ring. In the IR spectra of structurally similar complexes *trans*-[PdCl_2_(7AI3CA)_2_] and *trans*-[PtCl_2_(7AI3CA)_2_], the analogous NH stretching modes appear at similar frequencies, 3272 and 3193 cm^−1^, respectively [22,23]. The observed differences between the measured and calculated NH stretching frequencies in the title complexes suggest that the NH group may participate in intermolecular interactions with the chloride ligand (Cl) of the neighboring molecule, similar to those seen in the crystal structure of *trans*-[PtCl_2_(7AI3CA)_2_] [22].

In the Raman spectrum of the Pd(II) complex with 5ClL, two strong bands appear at 1680 cm^−1^ and 1671 cm^−1^. The latter band is assigned to the C8=O1 aldehyde stretching vibration, and it corresponds to the calculated frequency of 1720 cm^−1^, while the former one is due to Fermi resonance. For the Pt(II) complex with 5ClL, a similar band pattern is observed with the bands at 1680 cm^−1^ and 1672 cm^−1^, attributed to Fermi resonance and the C8=O1 aldehyde stretching vibration, respectively. In contrast to *trans*-[PdCl_2_(5ClL)_2_], the Pd(II) complex with 4ClL shows only a single strong Raman band at 1679 cm^−1^, which corresponds to the C8=O1 stretch (calculated at 1719 cm^−1^). The differences between the measured and calculated frequencies suggest that the C8=O1 group in the complexes may be involved in intermolecular interactions, similar to those observed in the crystal structures of 7AICA-metal complexes [22].

The appearance of an additional band in the C=O stretching region for the 5ClL complexes is characteristic for the presence of *trans* configurations of the aldehyde group in the 5-chloro-substituted ligands.

Similar bands were reported earlier for the free ligands [24]. For example, in the Raman spectrum of 5ClL (*trans* conformer), two bands appear at 1663 cm^−1^ and 1681 cm^−1^, while for 4ClL (*cis* conformer), a single strong band is observed at 1654 cm^−1^ [24].

This trend is further supported by the spectra of the related compounds. In the Raman spectrum of the Pt(II) complex with unsubstituted 7-azaindole-3-carbaldehyde (which has the same orientation of the CHO group as 5ClL), two bands were observed at 1686 cm^−1^ (medium intensity) and 1665 cm^−1^ (very strong) [22]. Additionally, the Raman spectrum of 7-azaindole-3-carbaldehyde crystal (*trans* conformer) shows two strong bands at 1678 cm^−1^ and 1653 cm^−1^ [25]. Thus, the consistently observed characteristic doublet in the range between 1653 and 1681 cm^−1^ can be regarded as evidence for the presence of *trans* conformation of the CHO group in the studied compounds.

The coordination sphere around the metal centers plays a crucial role in shaping the vibrational spectra of complexes, especially in the range below 500 cm^−1^. The arrangement of ligands significantly affects vibrational modes related to the metal–ligand bonds. In *trans* isomers (e.g., *trans*-[MCl_2_(L)_2_]), the symmetric vibrations, ν_s_(M-Cl), are observed only in Raman spectroscopy, while antisymmetric vibrations, ν_as_(M-Cl), are detected only by IR spectroscopy (mutual exclusion rule). On the other hand, in *cis*-[MCl_2_(L)_2_] isomers, both the symmetric and antisymmetric (M-Cl) stretching vibrations can be seen in the IR and Raman spectra.

In the spectra of *trans*-[PdCl_2_(5ClL)_2_], the ν(Pd-Cl) bands appear at higher wavenumbers (353 cm^−1^ in IR and 302 cm^−1^ in Raman) compared to *trans*-[PdCl_2_(4ClL)_2_] (328 cm^−1^ in IR and 294 cm^−1^ in Raman). This difference suggests stronger Pd-Cl bonds in the former complex, likely due to electronic stabilization provided by the 5ClL ligand. A similar trend is observed when comparing *trans*-[PdCl_2_(5ClL)_2_] and *trans*-[PdCl_2_(7AI3CA)_2_] (in the latter complex, the ν(Pd-Cl) vibration was assigned at 344 cm^−1^ in IR and 300 cm^−1^ in Raman) [23]. Thus, the ν(Pd-Cl) stretching frequencies are also higher in the 5ClL complex, indicating stronger Pd-Cl bonding. The same pattern is observed in the spectra of Pt(II) complexes. The ν(Pt-Cl) stretching frequencies in *trans*-[PtCl_2_(5ClL)_2_] (344 cm^−1^ in IR and 331 cm^−1^ in Raman) are higher than those in *trans*-[PtCl_2_(7AI3CA)_2_] (332 cm^−1^ in IR and 325 cm^−1^ in Raman) [22]. These results support the conclusion that the 5ClL ligand provides enhanced stabilization and a strengthening of the M-L bonds in both Pd(II) and Pt(II) complexes.

According to calculations for *trans*-[PdCl_2_(4ClL)_2_], the ν(Pd-N7) stretching vibrations are coupled with the 4ClL ligand vibrations and contribute to the bands observed at 254 cm^−1^ (IR) and 247 cm^−1^ (Raman). The calculated frequencies for these modes (249 cm^−1^ for A_u_ and 245 cm^−1^ for A_g_) are very similar to the experimental values. For *trans*-[PdCl_2_(5ClL)_2_], the observed frequencies at 301 cm^−1^ (IR) and 285 cm^−1^ (Raman) for ν(Pd-N7) stretching vibrations indicate a stronger palladium–nitrogen bond in comparison to the *trans*-[PdCl_2_(4ClL)_2_] complex, further supporting the stabilizing effect of the 5ClL ligands.

In the vibrational spectra of *trans*-[PtCl_2_(5ClL)_2_], the ν(Pt-N7) modes are observed at 299 cm^−1^ (IR) and 294 cm^−1^ (Raman). The corresponding ν_as_(Pt–N7) and ν_s_(Pt–N7) stretching modes in *trans*-[PtCl_2_(7AI3CA)_2_] exhibit strong coupling with other vibrations, contributing to several bands below 260 cm^−1^ [22].

### 2.3. Antiproliferative Activity of Investigated Palladium(II) and Platinum(II) Complexes Against Various Cell Lines

**Figure 5:**
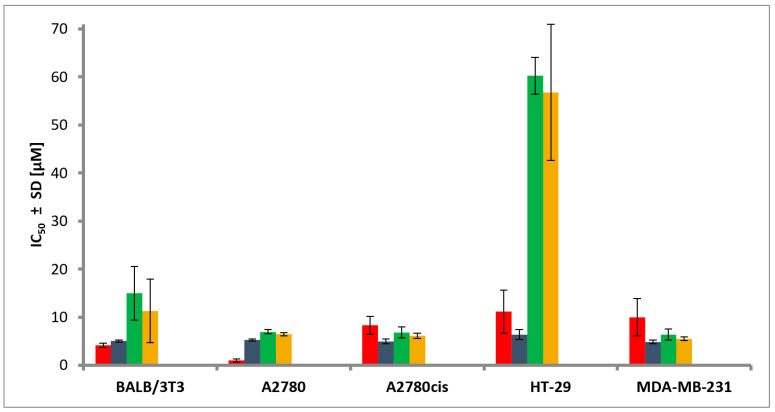
Antiproliferative activity of the investigated platinum(II) and palladium(II) complexes against various cell lines. Legend of colors: *cis*-[PtCl_2_(NH_3_)_2_] (cisplatin)—red, *trans*-[PtCl_2_(5ClL)_2_]—dark blue, *trans*-[PdCl_2_(5ClL)_2_]—green, *trans*-[PdCl_2_(4ClL)_2_]—yellow. The activity of the 4ClL and 5ClL ligands was not tested in the current study. Previous studies involving 7-azaindolo-3-carbaldehyde and halogenated 7-azaindole derivatives showed that these compounds did not exhibit antiproliferative activity on the tested cancer cell lines [20,22,23]. Based on these findings, it was assumed that the 4ClL and 5ClL ligands would exhibit a similar lack of activity.

The Pd(II) and Pt(II) complexes synthesized in this study were tested *in vitro* on several human cancer cell lines, including A2780 (ovarian cancer), A2780cis (cisplatin-resistant ovarian cancer), HT-29 (colon cancer), and MDA-MB-231 (triple-negative breast cancer), as well as on normal mouse fibroblast cells (BALB/3T3). Cisplatin was used as a reference compound. The obtained IC_50_ ± SD (µM) values are shown in Figure 5 and listed in Table S7 in the Supplementary Materials.

A lower IC_50_ value indicates higher antiproliferative activity, suggesting greater cytotoxic effectiveness against cancer cells. A higher IC_50_ value for healthy cells (e.g., BALB/3T3), may indicate better selectivity of the compound, leading to a lower risk of side effects while maintaining therapeutic efficacy.

The Selectivity Index (SI) is calculated as the ratio of the IC_50_ value for the normal cell line BALB/3T3 to the IC_50_ value for the corresponding cancer cell line. According to the literature, an SI value greater than 1.0 indicates that a compound exhibits greater antiproliferative activity against cancer cells than toxicity toward normal cells. Compounds with an SI value above 3.0 are considered highly selective [36].

The SI values for the compounds were calculated from the data presented in Table S7 in the Supplementary Materials. The SI values for *trans*-[PtCl_2_(5ClL)_2_] are as follows: 0.95 (A2780), 1.01 (A2780cis), 0.78 (HT-29), and 1.04 (MDA-MB-231). For *trans*-[PdCl_2_(5ClL)_2_], the SI values are 2.16 (A2780), 2.20 (A2780cis), 0.25 (HT-29), and 2.35 (MDA-MB-231). For *trans*-[PdCl_2_(4ClL)_2_], the SI values are 1.75 (A2780), 1.84 (A2780cis), 0.20 (HT-29), and 2.06 (MDA-MB-231). Finally, for *cis*-[PtCl_2_(NH_3_)_2_] (cisplatin), the SI values are 4.27 (A2780), 0.49 (A2780cis), 0.37 (HT-29), and 0.41 (MDA-MB-231).

The compound *cis*-[PtCl_2_(NH_3_)_2_] (cisplatin) exhibits the highest selectivity for the A2780 cell line (SI = 4.27), suggesting significant effectiveness in treating this cancer type while minimizing toxicity to normal cells. In contrast, *trans*-[PdCl_2_(5ClL)_2_] displays comparable selectivity for the A2780, A2780cis, and MDA-MB-231 cell lines, with SI values ranging from 2.16 to 2.35, but it shows substantially lower selectivity for the HT-29 cell line (SI = 0.25).

The complexes *trans*-[PdCl_2_(4ClL)_2_] and *trans*-[PdCl_2_(5ClL)_2_] showed comparable antiproliferative activity in the A2780 cell line, with IC_50_ values of 6.44 ± 0.37 µM and 6.94 ± 0.43 µM, respectively. These palladium complexes were less toxic to normal fibroblasts (BALB/3T3), with IC_50_ values of 11.29 ± 6.65 µM and 14.98 ± 5.59 µM, indicating better selectivity compared to the platinum complex. However, it is worth mentioning that *trans*-[PdCl_2_(4ClL)_2_] did not dissolve clearly in DMSO, unlike the platinum and palladium complexes with 5ClL.

In the A2780cis ovarian cancer cell line (which is resistant to cisplatin), cisplatin exhibited moderate antiproliferative activity (IC_50_ = 8.34 ± 1.86 µM), suggesting the development of resistance. Interestingly, both the *trans*-[PtCl_2_(5ClL)_2_] complex and its palladium analogs retained activity against this cell line, with IC_50_ values of 4.96 ± 0.49 µM for platinum, and 6.81 ± 1.17 µM and 6.13 ± 0.56 µM for *trans*-[PtCl_2_(5ClL)_2_] and *trans*-[PtCl_2_(4ClL)_2_], respectively. These findings suggest that the new complexes may be effective in treating cisplatin-resistant cancers.

*In vitro* tests using the HT-29 colon cancer cell line showed a lower activity for all the compounds compared to ovarian cancer cell lines. The most active compound was *trans*-[PtCl_2_(5ClL)_2_] (IC_50_ = 6.39 ± 1.07 µM), while the corresponding palladium complexes exhibited significantly higher IC_50_ values (60.25 ± 3.82 µM and 56.81 ± 14.17 µM), indicating limited efficacy against this type of cancer.

In the tests with the aggressive triple-negative breast cancer cell line MDA-MB-231, *trans*-[PtCl_2_(5ClL)_2_] showed moderate activity (IC_50_ = 4.83 ± 0.38 µM), while the palladium complexes displayed similar effectiveness (IC_50_ = 6.37 ± 1.19 µM for *trans*-[PtCl_2_(5ClL)_2_] and 5.48 ± 0.39 µM for *trans*-[PtCl_2_(4ClL)_2_]). Interestingly, cisplatin was less effective against this line (IC_50_ = 9.96 ± 3.89 µM), indicating that the platinum(II) and palladium(II) complexes may offer an advantage over cisplatin in treating triple-negative breast cancer.

When comparing the current results with the literature data for the unsubstituted complexes *trans*-[PtCl_2_(7AI3CA)_2_] [22] and *trans*-[PdCl_2_(7AI3CA)_2_] [23], significant differences in anticancer cytotoxicity can be observed.

In breast cancer cell lines, particularly MDA-MB-231, the *trans*-[PtCl_2_(5ClL)_2_] (IC_50_ = 4.83 ± 0.38 µM) showed significantly higher antiproliferative activity compared to the unsubstituted *trans*-[PtCl_2_(7AI3CA)_2_], which was much less active against the related MCF7 cell line (IC_50_ = 12.4 ± 5.8 µM) [22]. These results suggest that the incorporation of the chlorine atom into the ligand of the platinum complex significantly enhances its cytotoxic effectiveness in breast cancer models.

In colon cancer cell lines, specifically HT-29, the *trans*-[PtCl_2_(5ClL)_2_] (IC_50_ = 6.39 ± 1.07 µM) also demonstrated better antiproliferative activity than the unsubstituted *trans*-[PtCl_2_(7AI3CAH)_2_], which was less active against the related LoVo line (IC_50_ = 8.1 ± 1.5 µM) [22]. This indicates that chloro-substitution in the ligand of the platinum complex enhances its cytotoxicity in colon cancer models.

For palladium complexes, the known unsubstituted *trans*-[PdCl_2_(7AI3CA)_2_] showed similar or slightly higher activity in ovarian cancer cells A2780 (IC_50_ = 3.7 ± 2.9 µM) [23] compared to its chloro-substituted *trans*-[PdCl_2_(5ClL)_2_] (IC_50_ = 6.94 ± 0.43 µM). However, in normal fibroblast cells (BALB/3T3), the chloro-substituted complex *trans*-[PdCl_2_(5ClL)_2_] was significantly less toxic (IC_50_ = 14.98 ± 5.59 µM, SI = 2.16) than the unsubstituted *trans*-[PdCl_2_(7AI3CA)_2_] (IC_50_ = 4.1 ± 2.5 µM, SI = 1.11) [23], suggesting that chloro-substitution of the 7-azaindole-3-carbaldehyde ligand enhances selectivity of metal complexes by reducing toxicity to normal cells.

These findings highlight the potential of these new complexes, particularly their increased selectivity toward ovarian cancer cell lines (A2780) and enhanced effectiveness against cisplatin-resistant cancers and triple-negative breast cancer.

### 2.4. Stability of Trans-[PtCl_2_(5ClL)_2_] and Trans-[PdCl_2_(5ClL)_2_] in DMSO Solution

To investigate the stability of *trans*-[PtCl_2_(5ClL)_2_] and *trans*-[PdCl_2_(5ClL)_2_] in DMSO solution, we recorded their MIR (ATR) spectra at various time intervals: immediately after mixing (0 h) and then after 2, 24, 48, and 72 h. The results are illustrated in Figures S1 and S2 in the Supplementary Materials.

In the spectra of solid *trans*-[PtCl_2_(5ClL)_2_] and *trans*-[PdCl_2_(5ClL)_2_], the characteristic bands at 3319 cm^−1^ and 3318 cm^−1^, respectively, correspond to the ν(N1–H) stretching vibration of the pyrrole group in the 5ClL ligand. Upon dissolution of complexes in DMSO, these bands disappear, and a broad band appears between 2900 and 2700 cm^−1^, indicating the formation of strong N–H⋯O=S hydrogen bonds between the pyrrole NH group and DMSO. This is further confirmed by the shift of the ν(S=O) band from 1042 cm^−1^ (pure DMSO) to 1017–1018 cm^−1^ (start), 1021 cm^−1^ (2 h), 1021–1022 cm^−1^ (24 h), 1025–1021 cm^−1^ (48 h), and 1020–1022 cm^−1^ (72 h) for both complexes. Beyond 72 h, no further significant changes were observed, indicating that the complexes remained stable in solution. Similar shifts were observed previously for *trans*-[PtCl_2_(7AI3CA)_2_] [22] and *trans*-[PdCl_2_(7AI3CA)_2_] [23] in DMSO solution, supporting the conclusion of hydrogen bond formation between the solvent and the pyrrole group of the organic ligand in the complexes. These results demonstrate that *trans*-[PtCl_2_(5ClL)_2_] and *trans*-[PdCl_2_(5ClL)_2_] are stable in DMSO solution during the time of the tests of antiproliferative activity. However, we were unable to perform similar stability tests for *trans*-[PdCl_2_(4ClL)_2_] due to its poor solubility in DMSO.

## 3. Materials and Methods

### 3.1. Preparation of Trans-[PdCl_2_(4ClL)_2_] and Trans-[MCl_2_(5ClL)_2_] (M = Pd(II), Pt(II))

The complexes *trans*-[PdCl_2_(4ClL)_2_] and *trans*-[MCl_2_(5ClL)_2_] (M = Pd(II), Pt(II)) were synthesized following a modified procedure for the synthesis of *trans*-[PtCl_2_(7AI3CA)_2_] and *trans*-[PdCl_2_(7AI3CA)_2_] as described in references [22,23].

In this process, aqueous solutions of K_2_PtCl_4_ or K_2_PdCl_4_ (Sigma-Aldrich, Burlington, MA, USA) were combined with ethanol solutions of ligands 5ClL or 4ClL (Ambeed, Inc., Arlington Heights, IL, USA) in a 1:2 molar ratio. The reaction mixtures were stirred at 318 K for 48 h. Then, precipitates were filtered, washed with distilled water and ethanol, and dried.

The results from elemental analysis for these complexes, including experimental and theoretical values for carbon, hydrogen, and nitrogen content, are provided in Table S8 in the Supplementary Materials.

### 3.2. Spectroscopic Measurements

Fourier transform mid-infrared (MIR) and far-infrared (FIR) spectra were recorded using a Bruker VERTEX 70 V spectrometer (Bruker Optics GmbH & Co. KG, Ettlingen, Germany) equipped with a diamond ATR accessory and an air-cooled DTGS detector. The spectrometer was kept under vacuum, under a pressure below 1 hPa, and spectra were obtained at a resolution of 2 cm^−1^ with 64 scans averaged. ATR spectra were processed into absorbance spectra using OPUS™ 7.5 software. The transformation of the reflectance spectrum (R) to an absorbance spectrum (A) was performed using a simple logarithmic relationship without applying any correction for the thickness of the layer from which the ATR-FTIR spectrum was obtained.

ATR (MIR) spectra of a 0.5% solution of *trans*-[PtCl_2_(5ClL)_2_] and *trans*-[PdCl_2_(5ClL)_2_] in DMSO were measured. Initially, the concentration of the solutions was 2.0%. However, the complexes did not fully dissolve. Therefore, ultrasonic treatment was applied, and the solutions were diluted to a final concentration of 0.5%.

The spectra were recorded immediately after complete mixing (approximately 5 min), then after 2, 24, 48, and 72 h. For each measurement, a drop of the solution was placed on the surface of the ATR adapter. Spectra were collected at a resolution of 2 cm^−1^ with 64 scans averaged.

The FT-Raman spectra (3500–50 cm^−1^ range) were recorded on a Bruker MultiRAM spectrometer (Bruker Optics GmbH & Co. KG, Ettlingen, Germany) equipped with a liquid-nitrogen-cooled germanium detector and a Nd:YAG laser (1064 nm). Spectra were recorded at a resolution of 4 cm^−1^ with an average of 256 scans. For the *trans*-[PtCl_2_(5ClL)_2_] sample, the laser power was set at 200 mW, while for the other two samples, it was set at 300 mW. The diameter of the measured area was up to 5 mm.

### 3.3. Theoretical Methods

Computational investigations were carried out using Gaussian 16 [37] within the density functional theory (DFT) framework, employing the ωB97X-D long-range corrected hybrid functional with dispersion correction [38]. For hydrogen, carbon, nitrogen, and oxygen atoms, we employed the 6–31++G(d,p) basis set, while for platinum and palladium atoms, the LanL2DZ effective core potential basis set was used [39,40,41]. Initial geometries for these computations were derived from X-ray diffraction (XRD) data of *trans*-[PtCl_2_(7AI3CA)_2_] [22], providing the reliable starting point for accurate calculations. Full optimization of each molecular structure was performed, and all convergence conditions of forces and displacements (averaged and maxima) were fulfilled. Calculations of vibrational frequencies for each molecular structure were carried out to test the nature of the obtained stationary point. No imaginary frequencies were obtained, which proves that the calculated geometries correspond to the minima on the potential energy surfaces. Frequencies were scaled as follows: 0.948 for frequencies ≥ 2000 cm^−1^, 0.953 for frequencies in the range of 1000–2000 cm^−1^, and 0.970 for frequencies < 1000 cm^−1^, following standard recommendations [42]. To assist in vibrational mode assignments, potential energy distributions (PEDs) were calculated using the FCART program (version 7.0) [43]. Normal modes were visualized graphically with Chemcraft software [44].

### 3.4. Biological Studies

The following human cancer cell lines were used for *in vitro* evaluation of antiproliferative activity: ovarian cancer (A2780), cisplatin-resistant ovarian cancer (A2780cis), colon cancer (HT-29), and triple-negative breast cancer (MDA-MB-231), along with normal murine fibroblasts (BALB/3T3 clone A31). These cell lines were obtained from the Hirszfeld Institute of Immunology and Experimental Therapy, PAS, Wroclaw, Poland. The A2780 and A2780cis cell lines were sourced from the European Collection of Authenticated Cell Cultures (ECACC), and the remaining cell lines were from the American Type Culture Collection (ATCC).

A2780 cells were cultured in an RPMI + GLUTAMAX medium (GIBCO, Thermo Fisher Scientific, Waltham, MA, USA) with 10% fetal bovine serum (FBS) (HyClone, Cytiva, Marlborough, MA, USA). A2780cis cells were cultured in an RPMI + GLUTAMAX medium (GIBCO) with 10% FBS (HyClone) and 0.3 µg/mL cisplatin (cisplatin 1 mg/mL Concentrate for Solution for Infusion, Accord Healthcare Polska, Pabianice, Poland). HT-29 cells were maintained in RPMI + opti-MEM (1:1) medium (RPMI from IITD PAN, Wrocław, Poland; opti-MEM from GIBCO, Thermo Fisher Scientific, USA), supplemented with 5% FBS (HyClone) and 2 mM glutamine (Sigma-Aldrich, Darmstadt, Germany). MDA-MB-231 cells were cultured in RPMI with glutamine (Biowest, Nuaillé, France) and 10% FBS (Sigma-Aldrich, Germany). BALB/3T3 fibroblasts were cultured in DMEM (high glucose) medium (Biowest, France) with 10% FBS (HyClone) and 2 mM L-glutamine (Sigma-Aldrich). All media contained 100 mg/mL streptomycin (Sigma-Aldrich, Germany) and 100 U/mL penicillin (Polfa Tarchomin, Warszawa, Poland). The cells were cultured in a humidified 5% CO_2_ atmosphere at 37 °C.

Test solutions of the compounds were freshly prepared for each experiment by dissolving 1 mg of the compound in 100 μL of DMSO and 900 μL of culture medium, yielding a 1 mg/mL concentration. For further dilutions, RPMI + opti-MEM (1:1) medium (RPMI with glutamine from Biowest, France; opti-MEM from GIBCO) supplemented with 5% FBS (HyClone) was used. The compounds were tested at concentrations of 100, 10, 1, and 0.1 μg/mL. Cisplatin was used as a control at 10, 1, 0.1, and 0.01 μg/mL, with corresponding solvent concentrations of 1, 0.1, 0.01, and 0.001%. To improve solubility, compounds were heated at 37 °C for 10–15 min after adding the solvent. The *trans*-[PdCl_2_(4ClL)_2_] solution remained turbid after dissolution, while solutions of the other compounds were clear.

Antiproliferative activity was assessed using the sulforhodamine B (SRB) assay, as described by Skehan et al. [45]. The tests were performed after 72 h of incubation with the compounds in 96-well plates. Absorbance was measured at 540 nm using a Synergy H4 Hybrid Reader (BioTek, Winooski, VT, USA). Each experiment included three technical replicates per concentration, with the tests repeated three to four times.

## 4. Conclusions

This study presents a comprehensive analysis of the structural and vibrational characteristics of metal complexes of 4-chloro and 5-chloro derivatives of 7-azaindole-3-carbaldehyde (*trans*-[PdCl_2_(4ClL)_2_] and *trans*-[MCl_2_(5ClL)_2_], where M = Pd(II) or Pt(II)).

The study findings highlight how the ligand conformations influence the stability and vibrational properties of these complexes. DFT calculations using the ωB97X-D method were employed to assess the energy differences (Δ) between the structures of complexes constructed from two *cis* or two *trans* ligand conformers. Spectroscopic data confirmed theoretical predictions not only for specific ligand conformers but also for the presence of *trans* isomers of metal complexes.

For *trans*-[PdCl_2_(4ClL)_2_], the structure constructed from the *cis* conformers of the 4ClL ligand is more stable than that built from the *trans* conformers, with an energy difference of approximately 4–5 kcal/mol. This preference for the *cis* conformer is due to reduced steric repulsion between the oxygen and chlorine atoms. In contrast, for the 5ClL complexes with Pd(II) and Pt(II), the *trans* conformers are favored due to lower steric hindrance, with Δ values of about 4 kcal/mol.

Detailed vibrational analysis has enabled us to make a distinction between the *cis* and *trans* ligand conformers present in the studied complexes. The presence of *trans* configurations of the aldehyde group in the 7-azaindole-3-carbaldehyde ligand (7AI3CA) [25] and in the 5-chloro derivative (5ClL) [24], as well as in metal complexes with these ligands, is confirmed by the Raman spectra of these compounds, where an additional band appears in the C=O stretching region. Such a band is not observed for the *cis* conformer of the 4ClL ligand [24], nor for its complex *trans*-[PdCl_2_(4ClL)_2_] (this work).

Both the ν(Pd-Cl) and ν(Pd-N) stretching frequencies are higher in *trans*-[PdCl_2_(5ClL)_2_] compared to the 4ClL complex, signaling stronger Pd-Cl and Pd-N bonds, in the former compound. The IR and Raman spectra of the investigated complexes confirm the *trans* arrangement of the ligands.

All the studied complexes demonstrate promising antiproliferative activity, particularly against ovarian cancer (including the cisplatin-resistant strains) and triple-negative breast cancer cell lines. The complexes *trans*-[PtCl_2_(5CIL)_2_], *trans*-[PdCl_2_(5CIL)_2_], and *trans*-[PdCl_2_(4CIL)_2_] showed effective activity against A2780 ovarian cancer cells, with the IC_50_ values comparable to cisplatin, and maintained their activity against cisplatin-resistant A2780cis cells. For triple-negative breast cancer (MDA-MB-231), the complexes exhibited better activity than cisplatin, suggesting a potential advantage in treating resistant cancers. The complexes also displayed selective cytotoxicity towards cancer cells, with reduced toxicity to normal fibroblasts (BALB/3T3), supporting their potential as targeted anticancer agents. The presence of chloro-substituted 7-azaindole-3-carbaldehyde ligands in metal complexes significantly enhances the antiproliferative activity compared to the analogous complexes with an unsubstituted ligand, particularly in breast cancer and colon cancer models.

## Data Availability

The original contributions presented in this study are included in the article or Supplementary Materials, and further inquiries can be directed to the corresponding author.

## Supplementary Materials

The following supporting information can be downloaded at: https://www.mdpi.com/article/10.3390/molecules29245909/s1, Table S1: Electronic energies and their relative values Δ [kcal/mol] for trans-[PdCl_2_(4ClL)_2_], calculated using the ωB97X-D method; Table S2: Electronic energies and their relative values Δ [kcal/mol] for trans-[MCl_2_(5ClL)_2_], calculated using the ωB97X-D method; Table S3: Comparison of calculated bond lengths (Å) and angles (°) for the most stable forms of *trans*-[PdCl_2_(4ClL)_2_] (Figure 1a), *trans*-[PdCl_2_(5ClL)_2_], and *trans*-[PtCl_2_(5ClL)_2_] (Figure 1d); Table S4: Experimental bands (FT-IR, FT-Raman) of *trans*-[PdCl_2_(4ClL)_2_] and theoretical wavenumbers ($\tilde{\nu }$^a^, cm^−1^) calculated for the structure shown in Figure 1a using the ωB97X-D method and 6-31++G(d,p)/LanL2DZ basis sets; Table S5: Experimental bands (FT-IR, FT-Raman) of *trans*-[PdCl_2_(5ClL)_2_] and theoretical wavenumbers ($\tilde{\nu }$^a^, cm^−1^) calculated for the structure shown in Figure 1d using the ωB97X-D method and 6-31++G(d,p)/LanL2DZ basis sets; Table S6: Experimental bands (FT-IR, FT-Raman) of *trans*-[PtCl_2_(5ClL)_2_] and theoretical wavenumbers ($\tilde{\nu }$^a^, cm^−1^) calculated for the structure shown in Figure 1d using the ωB97X-D method and 6-31++G(d,p)/LanL2DZ basis sets; Table S7: Antiproliferative activity of *trans*-[PtCl_2_(5CIL)_2_], *trans*-[PdCl_2_(5CIL)_2_], *trans*-[PdCl_2_(4CIL)_2_], and *cis*-[PtCl_2_(NH_3_)_2_] (cisplatin); Table S8: Experimental (exp.) and theoretical (theor.) elemental compositions of the synthesized complexes; Figure S1: The MIR spectra of DMSO (red line), *trans*-[PtCl_2_(5ClL)_2_] in the solid state (solid black line), and *trans*-[PtCl_2_(5ClL)_2_] in the DMSO solution after: start (gray), 2 h (green), 24 h (violet), 48 h (grenade), and 72 h (orange); Figure S2: The MIR spectra of DMSO (red line), *trans*-[PdCl_2_(5ClL)_2_] in the solid state (solid black line), and *trans*-[PdCl_2_(5ClL)_2_] in the DMSO solution after: start (gray), 2 h (green), 24 h (violet), 48 h (grenade), and 72 h (orange).
