# Supplementary material for: Vibrational and DFT Studies and Anticancer Activity of Novel Pd(II) and Pt(II) Complexes with Chloro Derivatives of 7-Azaindole-3-Carbaldehyde
Source: Molecules. 2024 Dec 14;29(24):5909. doi: 10.3390/molecules29245909 (PMC11678368; doi:10.3390/molecules29245909)
Supplement: Supplementary file 1 [file molecules-29-05909-s001.zip › molecules-3357278-supplementary.pdf]

APPENDIX. SUPPLEMENTARY MATERIALS

Vibrational and DFT Studies and Anticancer Activity of Novel Pd(II) and Pt(II) Complexes with Chloro Derivatives of 7-Azaindole-3-carbaldehyde

Ksenia Szmigiel-Bakalarz [1], Dagmara Kłopotowska [2], Joanna Wietrzyk [2], Magdalena Malik [3] and Barbara Morzyk-Ociepa [1,*]

[1] Institute of Chemistry, Faculty of Science and Technology, Jan Dlugosz University in Czestochowa, Armii Krajowej 13/15, 42-200 Czestochowa, Poland

[2] Hirszfeld Institute of Immunology and Experimental Therapy, Polish Academy of Sciences, 12 Rudolf Weigl St., 53-114, Wrocław, Poland

[3] Faculty of Chemistry, Wroclaw University of Science and Technology, Wybrzeże Wyspiańskiego 27, 50-370 Wroclaw, Poland

Keywords: 4-chloro-7-azaindole-3-carbaldehyde; 5-chloro-7-azaindole-3-carbaldehyde; platinum(II) complexes; palladium(II) complexes; IR spectroscopy; Raman spectroscopy; DFT; antiproliferative activity

* Correspondence: bmorzyk-ociepa@o2.pl

CONTENTS

Table S1. Electronic energies and their relative values Δ [kcal/mol] for *trans*-[$PdCl_2(4ClL)_2$], calculated using the ωB97X-D method.

Table S2. Electronic energies and their relative values Δ [kcal/mol] for *trans*-[$MCl_2(5ClL)_2$], calculated using the ωB97X-D method.

Table S3. Comparison of calculated bond lengths (Å) and angles (°) for the most stable forms of *trans*-[$PdCl_2(4ClL)_2$] (Figure 1a), *trans*-[$PdCl_2(5ClL)_2$], and *trans*-[$PtCl_2(5ClL)_2$] (Figure 1d).

Table S4. Experimental bands (FT-IR, FT-Raman) of *trans*-[$PdCl_2(4ClL)_2$] and theoretical wavenumbers ($\tilde{\nu}^a$, cm^{-1}) calculated for the structure shown in Figure 1a using the ωB97X-D method and 6-31++G(d,p)/LanL2DZ basis sets.

Table S5. Experimental bands (FT-IR, FT-Raman) of *trans*-[$PdCl_2(5ClL)_2$] and theoretical wavenumbers ($\tilde{\nu}^a$, cm^{-1}) calculated for the structure shown in Figure 1d using the ωB97X-D method and 6- 31++G(d,p)/LanL2DZ basis sets.

Table S6. Experimental bands (FT-IR, FT-Raman) of *trans*-[$PtCl_2(5ClL)_2$] and theoretical wavenumbers ($\tilde{\nu}^a$, cm^{-1}) calculated for the structure shown in Figure 1d using the ωB97X-D method and 6- 31++G(d,p)/LanL2DZ basis sets.

Table S7. Antiproliferative activity of *trans*-[$PtCl_2(5CIL)_2$], *trans*-[$PdCl_2(5CIL)_2$], *trans*-[$PdCl_2(4CIL)_2$] and *cis*-[$PtCl_2(NH_3)_2$] (cisplatin).

Table S8. Experimental (exp.) and theoretical (theor.) elemental compositions of the synthesized complexes.

Figure. S1. The MIR spectra of DMSO (red line), *trans*-[$PtCl_2(5ClL)_2$] in the solid state (solid black line) and *trans*-[$PtCl_2(5ClL)_2$] in the DMSO solution after: start (gray), 2h (green), 24h (violet), 48h (grenade), and 72h (orange).

Figure. S2. The MIR spectra of DMSO (red line), *trans*-[$PdCl_2(5ClL)_2$] in the solid state (solid black line) and *trans*-[$PdCl_2(5ClL)_2$] in the DMSO solution after: start (gray), 2h (green), 24h (violet), 48h (grenade), and 72h (orange).

Table S1. Electronic energies and their relative values Δ [kcal/mol] for *trans*-$[PdCl_2(4ClL)_2]$, calculated using the ωB97X-D method.

Structure	Energy Type	$E_{min.}$	Δ
trans-$[PdCl_2(4ClL)_2]$	$E_{min.}$	-1852698.97	4.66
	$E_{min.}$ + ZPE	-1852544.24	4.47

$E_{min.}$ represents the electronic energy at the minimum of the potential energy surface for structure 1a. ZPE denotes the zero point energy. Δ represents the energy difference between structures 1a and 1b, which are shown in Figure 1.

Table S2. Electronic energies and their relative values Δ [kcal/mol] for *trans*-$[MCl_2(5ClL)_2]$, calculated using the ωB97X-D method.

Structure	Energy Type	$E_{min.}$	Δ
trans-$[PtCl_2(5ClL)_2]$	$E_{min.}$	-1847945.17	4.42
	$E_{min.}$ + ZPE	-1847790.56	4.22
trans-$[PdCl_2(5ClL)_2]$	$E_{min.}$	-1852699.83	4.41
	$E_{min.}$ + ZPE	-1852545.30	4.19

$E_{min.}$ represents the electronic energy at the minimum of the potential energy surface for structure 1d. ZPE denotes the zero point energy. Δ represents the energy difference between structures 1d and 1c, which are shown in Figure 1.

Table S3. Comparison of calculated bond lengths (Å) and angles (°) for the most stable forms of *trans*-[$PdCl_2(4ClL)_2$] (Figure 1a), *trans*-[$PdCl_2(5ClL)_2$], and *trans*-[$PtCl_2(5ClL)_2$] (Figure 1d).

	***trans*-[$PdCl_2(4ClL)_2$]**	***trans*-[$PdCl_2(5ClL)_2$]**	***trans*-[$PtCl_2(5ClL)_2$]**
N1-C2	1.361	1.364	1.364
C2-C3	1.377	1.377	1.377
C3-C3a	1.445	1.441	1.441
C3a-C4	1.395	1.392	1.392
C4-C5	1.389	1.388	1.388
C5-C6	1.391	1.397	1.396
C6-N7	1.340	1.340	1.342
N7-C7a	1.340	1.340	1.342
N1-C7a	1.366	1.370	1.369
C7a-C3a	1.418	1.410	1.409
C3-C8	1.467	1.455	1.455
C8-O1	1.217	1.217	1.217
C8-H8	1.103	1.109	1.109
C4-Cl/C5-Cl	1.732	1.738	1.738
N1-H1	1.018	1.018	1.016
C2-H2	1.080	1.081	1.081
C4-H4/C5-H5	1.083	1.084	1.084
C6-H6	1.082	1.082	1.081
N7-M	2.075	2.075	2.058
M-Cl	2.349	2.349	2.363
C2-N1-C7a	108.97	108.63	108.65
N1-C7a-N7	126.00	126.92	126.99
C6-N7-C7a	116.21	116.47	116.41
N1-C7a-C3a	108.29	108.57	108.58
C7a-N1-H1	122.02	122.17	122.63
N7-C7a-C3a	125.70	124.50	124.43
C2-N1-H1	126.78	126.75	126.62
C3-C8-O1	122.59	123.74	123.73
N1-C2-C3	110.55	110.31	110.27
N1-C2-H2	121.54	120.48	120.48
C2-C3-C8	122.97	126.19	126.19
C3-C2-H2	127.91	129.21	129.25
C3a-C3-C8	131.00	127.52	127.51
C3a-C4-Cl/C3a-C4-H4	121.62	121.36	121.37
C2-C3-C3a	106.03	106.29	106.30
C5-C4-Cl/C5-C4-H4	119.31	122.26	122.29
C4-C3a-C7a	115.98	118.73	118.84
C4-C5-H5/C4-C5-Cl	120.67	120.43	120.44
C3-C3a-C4	137.87	135.08	134.97
C6-C5-Cl/ C6-C5-H5	119.41	118.10	118.03
C3-C3a-C7a	106.14	106.18	106.18
N7-C6-H6	116.39	116.91	116.77
C3a-C4-C5	119.06	116.37	116.34
C5-C6-H6	120.50	120.64	120.79
C4-C5-C6	119.91	121.47	121.53
O1-C8-H8	120.61	120.70	120.70
N7-C6-C5	123.08	122.42	122.42
C3-C8-H8	116.80	115.57	115.57

N7-M-Cl	91.51/88.49	91.41/88.59	91.43/88.57
N7-M-N7	180.00	180.00	180.00
C6-N7-M-Cl	133.06/46.94	133.36/46.64	131.30/48.70
C7a-N7-M-Cl	127.31/52.69	128.22/51.78	127.17/52.83

Table S4. Experimental bands (FT-IR, FT-Raman) of *trans*-[PdCl$_2$(4ClL)$_2$] and theoretical wavenumbers ($\tilde{\nu}^a$, cm^{-1}) calculated for the structure shown in Figure 1a using the ωB97X-D method and 6-31++G(d,p)/LanL2DZ basis sets.

FT-IR	FT-Raman	$\tilde{\nu}^a$, A_u/A_g	Assignment [b]
3307 sh			FR
3219 m		3366, 3365	ν(N1H)
3143 w		3137, 3137	ν(C2H)
		3108, 3108	ν(C6H), ν(C5H)
3091 m	3094 m	3092, 3092	ν(C5H), ν(C6H)
3044 w	3047 w		FR
2872 w	2874 s	2877, 2877	ν(C8H)
1678 vs	1679 s	1720, 1719	ν(C8O)
1600 w	1599 w	1606, 1605	ν(R6)
1585 s	1586 w	1588, 1588	ν(R6)
1517 s	1519 vs	1516, 1515	ν(R5), δ(C2H)
1481 w	1482 w	1478, 1477	ν(R6), δ(C5H)
1445 m	1451 s	1434, 1432	δ(N1H), ν(R5)
1415 w	1414 w	1403, 1402	ν(R5), δ(R5/R6), δ(N1H)
1398 s	1399 m	1383, 1383	δ(C8H)
1335 s	1337 w	1322, 1322	ν(R6), δ(C6H)
1307 s	1310 m	1295, 1295	ν(R6)
1291 m	1293 w	1278, 1277	ν(R5), ν(C3C8)
1255 w	1254 m	1237, 1237	δ(N1H), δ(C2H)
1207 s	1207 w	1187, 1186	ν(R6), δ(C5H)
1186 m			
1110 m	1115 w	1103, 1101	ν(R5/R6), δ(C2H)
	1081 w	1098, 1098	δ(C2H), δ(N1H), ν(R6), δ(C5H)
1061 w	1064 w	1046, 1046	ν(C3C8), δ(R5)
982 w	999 w	984, 984	γ(C8H)
965 s	967 m	965, 964	ν(C4Cl), δ(R6)
		945, 944	γ(C6H), γ(C5H)
		882, 882	γ(C2H)
879 s	881 m	872, 872	δ(R5)
824 s		811, 810	γ(C5H), γ(C6H)
782 s	783 m	776, 776	δ(C8O1), ν(C3C8)
	768 m	765, 764	τ(R5/R6)
690 w	688 m	689, 687	δ(R5/R6), γ(N1H)
662 w		645, 645	γ(N1H)
635 w		617, 615	τ(R5/R6)
601 vs	602 m	597, 597	τ(R6)
589 s		588, 584	τ(R5/R6)
568 s	563 m	565, 559	δ(R5/R6)
542 w	535 w	538, 530	τ(R5/R6)
466 m	462 w	460, 455	ν(C4Cl), δ(C4Cl)
363 s		357 (Au)	δ(C8O1), δ(R5/R6), δ(ClPdN7)
	360 w	351 (Ag)	ν(C3C8), δ(C8O1)
		346 (Au)	τ(R5/R6), δ(ClPdN7)
	331 w	322 (Ag)	τ(R5/R6), δ(ClPdN7)
328 s		321 (Au)	ν(PdCl)
284 w		278 (Au)	γ(C3C8), τ(R5/R6), ν(PdN7)
	294 vs	276 (Ag)	ν(PdCl)
		258 (Ag)	τ(R5/R6), δ(ClPdN7), γ(C3C8)

FT-IR	FT-Raman	$\tilde{\nu}^a$, A_u/A_g	Assignment [b]
254 vs		249 (Au)	ν(PdN7), δ(C4Cl), δ(R6)
	247 w	245 (Ag)	ν(PdN7), δ(C4Cl), δ(C3C8), δ(R5/R6)
		233, 232	τ(R5/R6), γ(C3C8)
		226 (Au)	ν(PdN7), δ(ClPdN7)
195 m		187 (Au)	τ(R5/R6), δ(N7PdN7), δ(ClPdCl)
173 w		168 (Au)	δ(ClPdCl), δ(ClPdN7)
		165 (Ag)	δ(C3C8)
		154 (Ag)	δ(ClPdN7)
		146 (Au)	δ(C3C8), γ(ClPdN7)
127 vs		141 (Au)	γ(C3C8)
		139 (Ag)	ν(PdN7), δ(PdN7)
	125 vs	123 (Ag)	γ(C3C8)
		117 (Au)	γ(C3C8)
		109 (Au)	δ(ClPdCl), δ(ClPdN7)
		105 (Ag)	τ(R5/R6), γ(ClPdN7)
		83 (Ag)	τ(R5/R6), γ(C3C8)
		78 (Au)	τ(R5/R6), γ(C3C8)
		48 (Ag)	τ(R6), γ(PdCl), γ(C4Cl)
		45 (Ag)	τ(R5/R6), γ(ClPdN7)
		28 (Au)	τ(R5/R6), γ(C4Cl)
		19 (Au)	τ(R5/R6), γ(C4Cl)
		15 (Au)	τ(R5/R6), γ(C3C8)

[a] The wavenumbers calculated by the ωB97X-D method and scaled, as shown in Section 3.3. [b] Assignments from PED calculated by FCART06 and verified by Chemcraft program. Abbreviations: br, broad; m, medium; s, strong; v, very; w, weak; sh, shoulder; ν, stretching; δ, in-plane bending; γ, out-of-plane bending; τ, torsion; 5R, five-membered ring; 6R, six-membered ring; FR, Fermi resonance.

Table S5. Experimental bands (FT-IR, FT-Raman) of *trans*-[$PdCl_2(5ClL)_2$] and theoretical wavenumbers ($\tilde{\nu}^a$, cm^{-1}) calculated for the structure shown in Figure 1d using the ωB97X-D method and 6- 31++G(d,p)/LanL2DZ basis sets.

FT-IR	FT-Raman	$\tilde{\nu}^a$, A_u/A_g	Assignment [b]
3318 m		3367, 3367	ν(N1H)
		3121, 3121	ν(C2H)
3101 w	3107 w	3107, 3107	ν(C6H)
	3088 w	3091, 3091	ν(C4H)
3024 w			FR
2846 w	2847 w	2806, 2805	ν(C8H)
2767 w	2766 w		FR
2701 w			FR
	1680 s		FR
1672 vs	1671 vs	1721, 1720	ν(C8O1)
1604 w	1604 w	1617, 1616	ν(R6)
1585 w	1586 w	1595, 1594	ν(R6)
1526 m	1527 s	1531, 1530	ν(R5), δ(C2H)
1463 m	1465 w	1464, 1462	ν(R5/R6)
1434 s	1441 m	1425, 1423	δ(N1H), ν(R5)
1396 w	1396 w	1410, 1408	ν(R5), δ(N1H)
1382 w	1382 w	1362, 1362	δ(C8H)
1357 w	1357 w	1340, 1340	ν(R6), δ(C6H)
1298 w	1299 w	1289, 1289	ν(R5/R6)
1277 s	1277 w	1272,1271	ν(R6)
1233 w	1228 m	1227, 1227	δ(C4H), ν(R6/R5)
1182 w		1218, 1218	δ(N1H), δ(C2H)
1135 m	1135 w	1119, 1118	ν(C5Cl), ν(R5/R6), δ(C2H)
1101 m	1100 w	1101, 1101	δ(C2H), ν(R5)
1080 w	1079 w	1066, 1064	δ(C4H), ν(R6)
	1003 w	979, 979	γ(C8H)
960 w		945, 944	γ(C4H)
919 m	918 m	922, 922	δ(R5), δ(R6)
880 m		890, 889	γ(C6H)
867 m	867 w	858, 857	γ(C2H)
836 w	813 s	813, 812	ν(R5/R6)
798 s		799, 799	δ(C8O1), δ(R5)
766 w	766 w	763, 760	τ(R5/R6), γ(C4H)
728 s	728 w	723, 722	ν(C5Cl, δ(R5/R6), δ(C8O1)
693 br		642, 640	γ(N1H)
632 m	622 w	625, 620	γ(C8O1), τ(R5)
585 vs	582 w	603, 596	γ(N1H), τ(R5/R6)
561 s	563 w	573, 573	τ(R5/R6)
491 w	503 m	497, 494	ν(C3C8), δ(C3C8)
469 w	469 w	492, 473	τ(R5/R6)
	458 w	460, 452	δ(R5/R6), δ(C8O1)
378 w	376 m	374, 373	ν(C5Cl), δ(R6)
353 s		338 (Au)	ν(PdCl)
332 m	334 w	332, 331	γ(C3C8), τ(R5/R6)
		318, 318	τ(R5/R6), γ(C3C8)
	302 s	272 (Ag)	ν(PdCl)
301 w		292 (Au)	ν(PdN7), δ(C5Cl)
	285 w	284 (Ag)	ν(PdN7), δ(C5Cl)

FT-IR	FT-Raman	$\tilde{\nu}^a$, A_u/A_g	Assignment [b]
229 m		219 (Au)	γ(C8O1), τ(R5/R6)
		211 (Au)	δ(ClPdN7), ν(PdN7), δ(C5Cl)
219 s	217 w	182, 204	γ(C3C8), τ(R5/R6)
	171 w	175 (Ag)	δ(ClPdN7)
179 w		171 (Au)	γ(ClPdN7)
165 w		158 (Au)	δ(ClPdN7)
		134 (Ag)	δ(C3C8)
147 w	145 s	131, 129	τ(R5/R6)
126 vs	126 vs	121, 118	δ(C3C8)
	101 vs	105 (Ag)	δ(ClPtN7), τ(R5/R6)
97 vs		106 (Au)	γ(PdCl)
		91 (Ag)	δ(ClPdN7)
		83 (Au)	τ(R5), γ(C8O1)
		53 (Ag)	γ(PdCl)
		44 (Ag)	τ(R5/R6), γ(PdCl)
		29 (Au)	τ(R5/R6)
		18 (Au)	τ(R5/R6)
		14 (Au)	τ(R5/R6)

[a] The wavenumbers calculated by the ωB97X-D method and scaled, as shown in Section 3.3. [b] Assignments from PED calculated by FCART06 and verified by Chemcraft program. Abbreviations: br, broad; m, medium; s, strong; v, very; w, weak; sh, shoulder; ν, stretching; δ, in-plane bending; γ, out-of-plane bending; τ, torsion; 5R, five-membered ring; 6R, six-membered ring; FR, Fermi resonance.

Table S6. Experimental bands (FT-IR, FT-Raman) of *trans*-$[PtCl_2(5ClL)_2]$ and theoretical wavenumbers ($\tilde{\nu}^a$, cm^{-1}) calculated for the structure shown in Figure 1d using the ωB97X-D method and 6- 31++G(d,p)/LanL2DZ basis sets.

FT-IR	FT-Raman	$\tilde{\nu}^a$, A_u/A_g	Assignment [b]
3319 m		3394, 3393	ν(N1H)
3101 w	3105 w	3121, 3121	ν(C2H)
	3087 w	3112, 3111	ν(C6H)
	3054 w	3092, 3092	ν(C4H)
2845 w	2846 w	2806, 2806	ν(C8H)
	2766 w		FR
	1680 s		FR
1674 vs	1672 vs	1721, 1721	ν(C8O1)
1603 w	1602 w	1618, 1616	ν(R6)
1588 w	1587 w	1598, 1598	ν(R6)
1526 m	1526 m	1532, 1531	ν(R5), δ(C2H)
1463 m	1465 w	1465, 1463	ν(R5/R6)
1434 s	1439 m	1425, 1423	δ(N1H), ν(R5)
1397 w	1396 m	1411, 1409	ν(R5), δ(N1H)
		1362,1362	δ(C8H)
1353 w	1351 w	1341, 1341	ν(R6), δ(C6H)
1298 w	1298 w	1289, 1288	ν(R5/R6)
1277 s	1274 w	1272, 1272	ν(R6)
1237 w	1228 m	1228, 1227	δ(C4H), ν(R5/R6)
		1217, 1217	δ(C2H), δ(C6H)
1138 w	1138 w	1121, 1118	ν(C5Cl), ν(R5/R6), δ(C2H)
1100 m	1099 w	1100, 1100	δ(C2H), ν(R5)
	1080 w	1067, 1066	δ(C4H), δ(C6H)
	1003 w	979, 979	δ(C8H)
972 w		945, 944	γ(C4H)
920 m	918 w	924, 923	δ(R5/R6)
882 m		892, 891	γ(C6H)
867 w	867 w	857, 857	γ(C2H)
	819 s	815, 811	ν(R5/R6)
799 s		798, 798	δ(C8O1), δ(R5)
765 w		769, 766	τ(R5/R6), γ(C4H)
734 s	738 w	727, 720	ν(C5Cl, δ(R5/R6), δ(C8O1)
638 m		636, 622	δ(R5/R6), γ(N1H)
627 vw	623 w	628, 625	γ(C8O1), τ(R5)
587 vs	588 w	589, 581	γ(N1H)
562 s	565 w	570, 568	τ(R5/R6)
506 w	502 w	507, 480	τ(R5/R6)
498 w		495, 494	ν(C3C8), δ(C3C8)
469 w	461 w	464, 456	δ(R5/R6), δ(C8O1)
378 w	376 w	374, 373	ν(C5Cl), δ(R6)
344 s		308 (Au)	ν(PtCl)
334 sh		332, 331	τ(R5/R6), γ(C3C8)
		325, 318	τ(R5/R6), γ(C3C8)
	331 m	296 (Ag)	ν(PtCl)
299 w		283 (Au)	ν(PtN7)
	294 w	285 (Ag)	ν(PtN7)
235 m		222 (Au)	τ(R5/R6), γ(C8O1)
	221 w	205 (Ag)	τ(R5/R6), γ(C8O1)

FT-IR	FT-Raman	$\tilde{\nu}^a$, A_u/A_g	Assignment [b]
198 m		183 (Au)	γ(C3C8), δ(ClPtN7)
165 w	172 w	178, 170	γ(C3C8), δ(ClPtN7)
150 m		168 (Au)	γ(ClPtN7)
140 m		153 (Au)	δ(ClPtN7
	148 m	136 (Ag)	δ(C3C8)
126 vs	129 m	131, 129	τ(R5/R6)
	118 m	121, 118	δ(C3C8)
	103 m	111 (Ag)	δ(ClPtN7), τ(R5/R6)
95 vs		109 (Au)	γ(PtCl)
	94 m	96 (Ag)	δ(ClPtN7)
		81 (Au)	τ(R5), γ(C8O1)
		54 (Ag)	τ(R5/R6), γ(PtCl)
		48 (Ag)	τ(R5/R6), γ(PtCl)
		29 (Au)	τ(R5/R6), δ(PtN7)
		19 (Au)	τ(R5/R6)
		15 (Au)	τ(R5/R6)

[a] The wavenumbers calculated by the ωB97X-D method and scaled, as shown in Section 3.3. [b] Assignments from PED calculated by FCART06 and verified by Chemcraft program. Abbreviations: br, broad; m, medium; s, strong; v, very; w, weak; sh, shoulder; ν, stretching; δ, in-plane bending; γ, out-of-plane bending; τ, torsion; 5R, five-membered ring; 6R, six-membered ring; FR, Fermi resonance.

Table S7. Antiproliferative activity of *trans*-$[PtCl_2(5CIL)_2]$, *trans*-$[PdCl_2(5CIL)_2]$, *trans*-$[PdCl_2(4CIL)_2]$ and *cis*-$[PtCl_2(NH_3)_2]$ (cisplatin).

Complexes	Cell lines */ IC_{50} ± SD [μM]				
	A2780	A2780cis	HT-29	MDA-MB-231	BALB/3T3
trans-$[PtCl_2(5CIL)_2]$	5.26 ± 0.21	4.96 ± 0.49	6.39 ± 1.07	4.83 ± 0.38	5.01 ± 0.27
trans-$[PdCl_2(5CIL)_2]$	6.94 ± 0.43	6.81 ± 1.17	60.25 ± 3.82	6.37 ± 1.19	14.98 ± 5.59
trans-$[PdCl_2(4CIL)_2]$	6.44 ± 0.37	6.13 ± 0.56	56.81 ± 14.17	5.48 ± 0.39	11.29 ± 6.65
cis-$[PtCl_2(NH_3)_2]$	0.96 ± 0.33	8.34 ± 1.86	11.13 ± 4.52	9.96 ± 3.89	4.09 ± 0.43

* Cell lines: A2780 (human ovarian carcinoma), A2780cis (cisplatin-resistant human ovarian carcinoma), HT-29 (human colon cancer), MDA-MB-231 (human mammary gland cancer), BALB/3T3 clone A31 (normal murine fibroblast).

Table S8. Experimental (exp.) and theoretical (theor.) elemental compositions of the synthesized complexes.

Complex	C (%) exp.	C (%) theor.	H (%) exp.	H (%) theor.	N (%) exp.	N (%) theor.
trans-$[PtCl_2(5ClL)_2]$	31.26	30.62	1.65	1.59	8.67	8.93
trans-$[PdCl_2(5ClL)_2]$	35.36	35.69	1.71	1.86	10.21	10.41
trans-$[PdCl_2(4ClL)_2]$	34.90	35.69	1.79	1.86	9.98	10.41

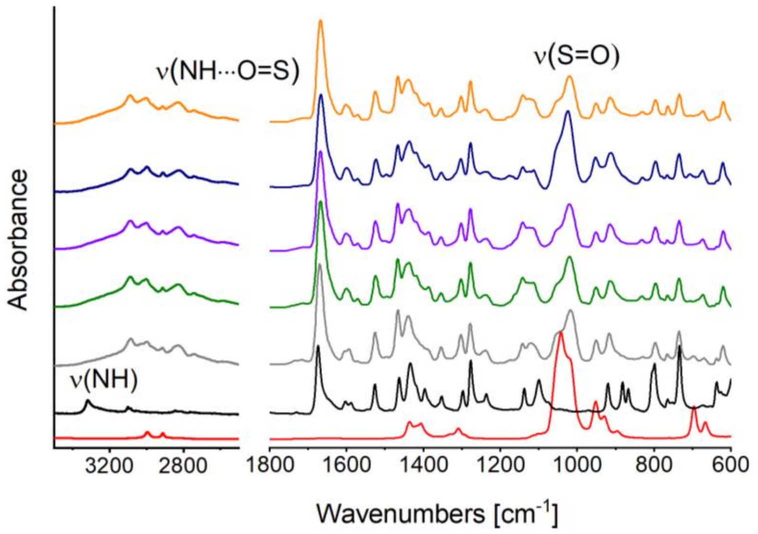

Figure. S1. The MIR spectra of DMSO (red line), *trans*-[PtCl2(5ClL)2] in the solid state (solid black line) and *trans*-[PtCl2(5ClL)2] in the DMSO solution after: start (gray), 2h (green), 24h (violet), 48h (grenade), and 72h (orange).

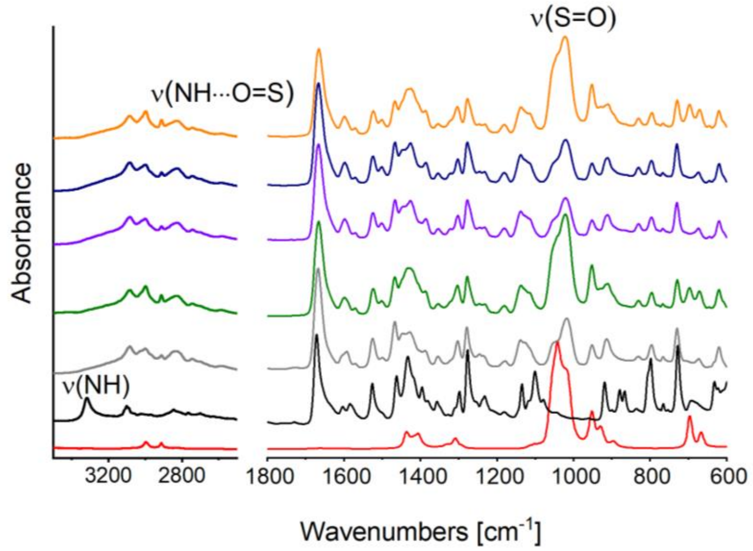

Figure. S2. The MIR spectra of DMSO (red line), *trans*-[PdCl2(5ClL)2] in the solid state (solid black line) and *trans*-[PdCl2(5ClL)2] in the DMSO solution after: start (gray), 2h (green), 24h (violet), 48h (grenade), and 72h (orange).
